# Multimodal Cardiac Imaging Revisited by Artificial Intelligence: An Innovative Way of Assessment or Just an Aid?

**DOI:** 10.7759/cureus.64272

**Published:** 2024-07-10

**Authors:** Marlon E Rivera Boadla, Nava R Sharma, Jeffy Varghese, Saral Lamichhane, Muhammad H Khan, Amit Gulati, Sakshi Khurana, Samuel Tan, Anupam Sharma

**Affiliations:** 1 Internal Medicine, Maimonides Medical Center, Brooklyn, USA; 2 Medicine, Manipal College of Medical Sciences, Pokhara, NPL; 3 Internal Medicine, NYC Health + Hospitals/Woodhull, Brooklyn, USA; 4 Internal Medicine, Gandaki Medical College, Pokhara, NPL; 5 Research, Maimonides Medical Center, Brooklyn, USA; 6 Cardiology, Icahn School of Medicine at Mount Sinai, New York, USA; 7 Radiology, Columbia University, Brooklyn, USA; 8 Internal Medicine, Icahn School of Medicine at Mount Sinai, New York, USA; 9 Hematology and Oncology, Fortis Hospital, Noida, IND

**Keywords:** artificial intelligence in healthcare, artificial intelligence in radiology, artificial intelligence in medicine, aritifical intelligence, deep learning artificial intelligence

## Abstract

Cardiovascular disease remains a leading global health challenge, necessitating advanced diagnostic approaches. This review explores the integration of artificial intelligence (AI) in multimodal cardiac imaging, tracing its evolution from early X-rays to contemporary techniques such as CT, MRI, and nuclear imaging. AI, particularly machine learning and deep learning, significantly enhances cardiac diagnostics by estimating biological heart age, predicting disease risk, and optimizing heart failure management through adaptive algorithms without explicit programming or feature engineering. Key contributions include AI's transformative role in non-invasive coronary artery disease diagnosis, arrhythmia detection via wearable devices, and personalized treatment strategies. Despite substantial progress, challenges including data standardization, algorithm validation, regulatory approval, and ethical considerations must be addressed to fully harness AI's potential. Collaborative efforts among clinicians, scientists, industry stakeholders, and regulatory bodies are essential for the safe and effective deployment of AI in cardiac imaging, promising enhanced diagnostics and personalized patient care.

## Introduction and background

Cardiovascular disease (CVD) has remained one of the leading causes of overall mortality and morbidity in the world. Evaluation of CVD entails several steps, including medical history, physical examination, significant family history, testing with laboratories, and imaging in some instances. With the evolving field of cardiology and new advances in therapy, provider decision-making has become more challenging. Nevertheless, introducing and developing new technologies have also provided a counterpart to ease the evaluation and prediction and aid decision-making [1]. The roots of multimodal imaging, a field that has significantly advanced cardiovascular diagnostics, can be traced back to 1895 when Roentgen captured the first radiation image of his wife’s hand [2]. Since then, the field has evolved from X-rays to computerized tomography (CT) scans, magnetic resonance imaging (MRI), and nuclear studies. These advancements, developed and refined over the past few decades, have provided a new perspective on diagnosing and advances in treatment and marked a milestone in the history of medical imaging, deserving our utmost respect and appreciation. 

Artificial intelligence (AI) was first introduced in the Second World War when Alan Turing designed the machine that would break the Enigma code at that time and wondered if, in the future, machines would be able to carry on different complex processes and, being one of them, the ability to rationalize [3]. Developing intricate machines that carry out increasingly complex processes has made current disciplines more manageable and specialized. Machine learning (ML) represents a facet of AI that operates without the necessity for explicit programming. In cardiac imaging studies, ML techniques find broad application, undertaking various tasks such as estimating biological heart age, predicting heart disease risk, and forecasting heart failure. Deep learning (DL), a subset of ML, emulates the workings of the human brain, exhibiting superior efficiency in executing simple and complex tasks compared to alternative systems. DL models dispense with the need for feature engineering, a requirement often associated with classical ML models [1,4]. 

Cardiovascular imaging alludes to different methods developed and is now used to study the heart's anatomy, function, physiology, and structure. X-rays, cardiac CT, cardiac MRI, and echocardiography are current techniques used to assess the heart, and each is used to evaluate different perspectives. Nuclear imaging is a developing field, and it is now being used more frequently due to its non-invasive nature and ability to offer cardiac functionality and physiology from different standpoints, now also being able to be interpreted by AI, posing ground for improvement in overall assessment [5]. Given AI's rapid evolution and its different modalities and branches, we aim to review the various imaging studies in which AI has aided cardiologists in finding alternate pathways, predicting outcomes, and guiding management in some instances.

## Review

The rapid integration of AI into healthcare, particularly within cardiac imaging, is underpinned by the exponential growth of healthcare datasets. With the digitization of medical records, imaging studies, genomic data, and wearable device information, there has been an unprecedented accumulation of vast and diverse healthcare data. This wealth of data serves as a cornerstone for training AI algorithms to perform myriad tasks, ranging from medical imaging interpretation to predictive analytics and personalized medicine. In cardiac imaging, AI, reliant on ML algorithms, has witnessed significant advancements facilitated by strides in computing power and data storage. These advancements have ushered in a new era of innovation, with AI poised to transform various cardiac imaging modalities, including nuclear cardiology, transthoracic echocardiography (TTE), and transesophageal echocardiography (TEE). 

In nuclear cardiology, AI-driven techniques such as single-photon emission computed tomography (SPECT) and positron emission tomography (PET) are revolutionizing the assessment of myocardial perfusion and predicting cardiovascular outcomes. With improved photon sensitivity and resolution, SPECT is approaching PET's performance, while PET's ability to detect photon pairs offers high-quality images with lower radiation exposure [6]. Recent studies have demonstrated the utility of AI in linking perfusion metrics from these modalities to major adverse cardiovascular events (MACEs) over long-term follow-ups, showcasing its potential in risk prediction and prognosis assessment [7,8]. Similarly, in TTE, AI enhances image quality and analysis by identifying subtle patterns within extensive datasets, thereby promoting standardization and reducing physician variability [9]. Through integration with electronic health records and pathology reporting systems, AI aids in diagnostics and treatment planning, automating measurements, and improving myocardial analysis efficiency and accuracy. However, the integration of AI into TEE remains in its nascent stages, primarily due to the complex and varied nature of TEE data [10]. Despite its pivotal role in managing peri-operative cardiac conditions and monitoring critical care patients, challenges persist in ensuring reliable results through AI-driven automation. 

Nevertheless, advancements in convolutional neural networks and robust dataset acquisition promise to overcome these challenges and advance TEE automation. AutoMAPSE, an AI-driven technology for automatic quantification of mitral annular plane systolic excursion (MAPSE) through TEE, exemplifies the potential of AI in enhancing cardiac imaging. By swiftly aggregating and averaging MAPSE measurements from all available heartbeats, AutoMAPSE surpasses traditional clinical practices, minimizing variability and enabling the detection of subtle changes in the left ventricular function [11]. 

Furthermore, AI's impact extends beyond imaging modalities like TTE and TEE, including coronary computed tomography angiography (CCTA), enhancing lesion detection, diagnostic speed, and accuracy. Through ML fractional flow reserve-CT (ML FFR-CT) and other AI-driven techniques, AI offers non-invasive and cost-effective alternatives for assessing significant coronary lesions, improving risk stratification, and decision-making in coronary heart disease (CHD) management [12]. Moreover, AI's ability to analyze pericardial fat and quantify fat attenuation index facilitates risk prediction and management of adverse cardiovascular events [13]. AI enhances diagnostic accuracy and consistency by integrating imaging data and extracting quantitative information, revolutionizing CHD management. Researchers have explored DL methods to automate the measurement of native T1 and extracellular volume fractions in cardiovascular magnetic resonance (CMR) imaging, achieving high segmentation accuracy with reduced motion artifacts. AI-driven contouring for accurate late gadolinium enhancement (LGE) quantification and myocardial Infarction (MI) detection in non-contrast cine images, along with automated atrial fibrosis segmentation to assess atrial fibrillation risk using convolutional neural networks (CNNs) and 3D U-net architecture, are exemplary examples of the revolutionary utility of AI in cardiovascular imaging [14].

TTE

Since the invention of ultrasound and its extrapolation to be used in medicine, there has been a better understanding of diseases and decreased invasive interventions. The use of ultrasound and its ability to delineate the anatomy of multiple organs has aided in diagnosing and further treating patients, given that nowadays, many procedures are performed through ultrasound. AI algorithms have revolutionized TTE by enhancing image analysis and interpretation capabilities. These algorithms can accurately identify anatomical anomalies, measure velocities, and calculate essential cardiac parameters such as ejection fraction (EF) and global longitudinal strain (GLS). Nevertheless, one of the limitations has always been that they are operator-dependent, and interpretation is subjective to the eyes of the person performing or reading the test [15]. With the introduction of AI and its reach, ultrasonography has been revolutionized due to its ability to analyze and approach the interpretation of cardiac imaging through ultrasound. One of its most important applications has been the ability to improve imaging, identify anomalies, and measure different velocities and distances that were performed before by humans [9].

He et al. conducted a study demonstrating that AI was non-inferior to human sonographers in assessing different parameters, such as EF, in both time and accuracy [16]. This blinded, randomized trial involved independent cardiologists who could not distinguish whether AI or a sonographer conducted the initial evaluation. Going beyond the relatively straightforward EF calculation, AI holds the potential to significantly simplify the computation of three-dimensional (3D) EF, stroke volume, and valvular area. Beginning with existing 3D full-volume heart models, these models are then adjusted for speckle tracking of the individual patient [16]. Salte et al. performed a study to assess LV function using an echocardiogram and deep learning to improve utility and reduce user variability. AI demonstrated lower test-retest variability compared to scenarios involving different readers (data set I: minimum detectable change (MDC) = 3.7 vs. 5.5, mean absolute difference = 1.4 vs. 2.1, respectively; data set II: MDC = 3.9 vs. 5.2, mean absolute difference = 1.6 vs. 1.9, all P < .05). Bias was observed in GLS measurements in 13 out of 24 test-retest scenarios involving different readers, with the most significant bias being 3.2 strain units. In contrast, AI measurements showed no bias [17]. 

TEE

While the utilization of AI in TTE is rapidly expanding, its application to TEE remains fully established. Automated analysis of TEE data is constrained due to the complexity and lack of structure in TEE images and dynamics, which exhibit significant variability across different views for evaluating cardiac structures. The adoption of AI in TEE is hindered by the intricate multi-view format of echocardiography and the unavoidable necessity for human intervention in image acquisition and interpretation [11]. 

In a study conducted by Yu et al., they evaluated a method to estimate left ventricular function by measuring mitral annular plane systolic excursion through AI. They found that it took less than three heartbeats to measure the mitral annular plane systolic excursion, had a low bias, and was more precise than manual measurements if the averaged heartbeats were higher [10]. The application of AI in TEE is still widely to be studied and implemented due to its different applications in real-time scenarios and critical decisions. Steffner et al. described the identification by DL through which they exposed DL to other videos and images to be identified and correctly classified. They found that the DL model could identify the eight most common TEE views, intraprocedural and intraoperative, with high efficiency and accuracy [18]. 

CT coronaries

CCTA offers comprehensive imaging of coronary artery branches, aiding in the analysis of diseased vessels. It supports cardiovascular risk assessment and treatment planning, providing predictive insights into cardiac events. Its noninvasive, convenient, and cost-effective nature makes CCTA an optimal screening tool for CHD [12]. AI has found application in diverse areas of cardiac CT imaging, seeking to enhance image resolution, automate image reconstruction processes, extract clinically relevant scores and measurements from contrast and non-contrast images, and predict patient outcomes. These applications extend beyond assessing the coronary arteries to encompass other cardiac structures. CCTA is a valuable tool for detecting coronary artery disease (CAD), but frequent scans can increase radiation exposure risks. Researchers are exploring strategies to minimize radiation doses while preserving image quality to mitigate this. However, solely focusing on dose reduction may compromise diagnostic accuracy. AI technology presents a promising solution, allowing for radiation dose reduction without compromising image fidelity in CCTA scans [12,19]. 

Brodoefel et al. found that body size significantly influenced CCTA image quality. Larger-bodied patients necessitated higher tube voltage and current to achieve comparable image quality to smaller-bodied patients. However, elevating these parameters inevitably raises radiation exposure for patients. AI diminishes radiation exposure by analyzing CT images from standard-dose phases to filter noise from low-dose phases while preserving image details [20]. Coronary artery calcium (CAC) represents a sign of coronary atherosclerosis characterized by a complex and controllable process. Calculated through CCTA, the coronary artery calcium score (CACS) predicts cardiac events in asymptomatic individuals. Those with a CACS exceeding 100 will likely benefit from lipid-lowering therapy, potentially reducing atherosclerotic cardiovascular disease events [21]. Determining the CACS traditionally involves a semi-automatic process, necessitating the outlining of contours or manual identification of calcium-containing objects. This method is often time-consuming, requiring significant intervention by physicians. However, AI algorithms can efficiently locate and segment vascular calcifications, automating the calculation of the calcification score. Subsequently, diagnostic physicians review the CACS, significantly expediting the diagnostic process [12]. AI can swiftly analyze CT images and compute the CACS, addressing the existing scarcity of medical expertise in this domain.

Atherosclerotic plaque accumulation in coronary arteries leads to stenosis, causing myocardial ischemia and infarction. Plaque volume indicates CHD severity and prognosis. Plaques are classified into calcified, noncalcified, and mixed types, each requiring tailored treatment [22]. Visual assessment of plaque and stenosis from CCTA images is vital but laborious and prone to error, especially for noncalcified and mixed plaques [19]. AI can analyze textures and structures using specific features in its model, automatically automating plaque analysis and stenosis rate assessment. This reduces the workload for imaging staff. Additionally, AI's integration of imaging data allows for the quick and automatic extraction of vulnerable plaques, aiding accurate decision-making based on specific anatomical features [12,19]. 

Kolosvalay et al. integrated radiomic parameters into eight ML algorithms. They trained the ML model using 75% of the dataset. In comparison, the remaining 25% underwent visual and histogram evaluations, comparing the results with the ML model's performance using the area under the curve (AUC) metric. Their study revealed that the ML model surpassed visual assessment in identifying advanced atherosclerotic lesions [23]. Tesche and Rosendael conducted a comparative analysis of ML risk scores against traditional CT risk scores, including the Agatston calcium score and segment involvement score (SIS), using AUC. Their findings indicated that the ML model improved risk stratification accuracy based on plaque-derived information [24]. The ML model exhibited significantly higher AUC values than conventional CT risk scores, demonstrating strong agreement between unstable plaque measurements and clinical parameters, including the Framingham Risk Score. ML effectively extracted comprehensive plaque information from CCTA scans, allowing for more precise risk assessments. AI offers swift quantification of emerging imaging biomarkers. The assessment of pericardial fat, indicating cardiometabolic risk, is feasible through standard cardiac CT scans. Studies have shown DL's capability to automate epicardial fat measurement accurately, correlating well with manual methods.

Moreover, changes in perivascular adipose tissue, reflective of coronary artery inflammation, can enhance cardiac risk evaluation compared to conventional risk factors [13]. Recent advancements have seen the application of radiometric techniques in evaluating the left ventricle (LV) myocardium to pinpoint areas of ischemia or infarction. In a study by Mannil et al., radionics analysis on low-dose non-contrast CT scans distinguished acute or chronic myocardial infarction from standard cases in 87 patients. Their ML model achieved sensitivity up to 86% and specificity up to 85%, surpassing human readers. Moreover, radio mic features are invaluable in automating CT myocardial perfusion imaging assessment [25]. Additionally, direct myocardial analysis aids in assessing the severity of coronary artery stenosis in CCTA [26]. 

MRI

AI is revolutionizing the field of medical imaging, particularly in CMR, with sophisticated methods for image acquisition, reconstruction, and analysis. These advancements profoundly impact clinical decision-making processes, as recent years have seen a variety of methods emerge to optimize CMR data capture, reconstruction, post-processing, and analysis. Alongside these developments, significant efforts have been made to craft AI-driven biomarkers for various cardiac ailments, promising improvements in diagnostic accuracy and treatment approaches. The digitalization of MRI signals and the rich diversity of contrast and parametric information within the images render this field ideally suited for many AI techniques. A recent study found a 573% increase in UK scans over a decade [27]. This demands more resources, including expert time and scan costs. Advanced CMR techniques like high-resolution imaging and MR-derived biomarkers require a cost-effective and time-efficient integration into clinical practice. AI holds great promise in this context, given its ability to expedite MRI scanning, streamline image post-processing and reporting, introduce innovative biomarkers, and integrate them into decision-making and predictive models. Improving the pace of image acquisition can offer added benefits for patients experiencing claustrophobia, anxiety, or difficulties following breath-holding instructions during scans. By accelerating scanning and post-processing and enabling automated analysis, AI can facilitate broader access to sustainable, faster, and more cost-effective CMR solutions, leading to enhanced patient care, especially in underserved areas [28]. Utilizing neural networks, researchers have applied innovative techniques to reconstruct data from rapidly acquired under-sampled MRI images spanning different sequences. A super-resolution CMR angiography framework, built upon deep learning principles, has successfully reconstructed low-resolution data obtained within a 50-second scanning window, with dimensions measuring 1.2 x 4.8 x 4.8mm³ [29]. 

Steeden et al. employed a subset of the convolutional neural network, specifically the 3D residual U-net, for super-resolution reconstruction on low-resolution 3D datasets of the whole heart in balanced steady-state free precession (bSSFP). This approach yielded diagnostic confidence and accuracy similar to high-resolution whole heart bSSFP scans in patients diagnosed with CHD [30]. Zhang et al. introduced an innovative AI-based virtual native enhancement (VNE) imaging technique. This approach utilizes CNNs to refine signal acquisition from native T1 mapping and cine imaging sequences, transforming them into images akin to LGE. VNE offers contrast-free and efficient tissue characterization, exhibiting robust agreement in quantifying tissue burden and delivering superior image quality compared to conventional LGE images [31]. In a recent study, researchers employed a deep fully CNN to devise an automated segmentation approach for quantifying tissue characteristics in native T1 mapping among hypertrophic cardiomyopathy patients. This innovation demonstrated robustness against inter-observer variability and reduced analysis time to less than a second [32]. 

CNN is also employed for automated phase velocity estimation and segmentation of four-dimensional flow datasets. Additionally, they aid in estimating global and segmental myocardial strain from displacement encoding with stimulated echoes (DENSE) images. Accurate CMR imaging requires precise acquisition, as recommended by SCMR guidelines. Due to limited dedicated technicians, studies explore automating and expediting CMR acquisition. Lu et al. introduced a learning-based algorithm to automate and accelerate this process. They manually segmented a LV 3D model, co-registered it to the patient's heart using a tree-based classifier, and adjusted it to match the patient's anatomy [33]. The manual segmentation of cardiac volumes, involving tracing endocardial and epicardial contours, is crucial for evaluating biventricular function [34]. However, this process is time-consuming and becomes more challenging with variations in heart shape or datasets with low contrast-to-noise ratio. Various AI models have emerged to automate this segmentation, aiming to simplify, expedite, and enhance accuracy in this essential task. During the 20th International Conference on Medical Image Computing and Computer-Assisted Intervention (MICCAI), the "Automatic Cardiac Diagnosis Challenge" (ACDC) was held to determine the optimal AI model for automatic cardiac segmentation. Bernard et al. examined various deep-learning algorithms for segmentation and classification tasks [35]. Their results showcased a correlation score of 0.97 for the top-performing algorithm, demonstrating strong performance in left ventricle segmentation but falling short in evaluating the right ventricle and myocardium. LGE imaging and T1 and T2 mapping techniques enable the visualization and quantification of whether focal or diffuse myocardial disease. Native T1 mapping techniques are adept at detecting increased extracellular compartments observed in conditions such as amyloidosis, acute inflammation, or myocardial fibrosis, as well as identifying iron infiltration or Fabry's disease [36]. Elevated T2 mapping values provide highly accurate indications of myocardial edema. 

AI holds substantial promise in precisely evaluating these parameters, which is critical for diagnostic and prognostic purposes. In their investigation, Moccia et al. assessed a segmentation model employing a full CNN for LGE. They reported a Dice similarity coefficient of 71.3%, along with sensitivity, specificity, and accuracy rates of 88.1%, 97.9%, and 96.8%, respectively. AI can enhance risk stratification and prognosis prediction for patients with cardiomyopathies or undergoing invasive treatments [37]. Dawes et al. investigated using an ML survival model based on 3D cardiac motion to predict outcomes in patients with pulmonary hypertension, irrespective of conventional risk factors [38]. 

Stress test

Exercise stress testing is a foundational non-invasive diagnostic tool; however, its accuracy can vary based on age, gender, and clinical characteristics, prompting the exploration of more reliable methods. Recent advancements in ML, including DL and natural language processing, exhibit the potential to enhance the interpretation of stress-testing data. DL has shown utility in examining resting electrocardiograms (ECGs) and detecting arrhythmias like atrial fibrillation and ventricular tachycardia. Moreover, these algorithms have identified patterns suggestive of various cardiac conditions, including valvular diseases, cardiac amyloidosis, and hypertrophic cardiomyopathy. DL can also be applied to analyzing stress ECG and echocardiography [39]. Several investigations have showcased impressive performance metrics in ML-assisted stress echocardiography interpretation. 

Upton et al. devised an automated pipeline utilizing CNNs to extract features from stress echocardiography exams. These features were employed to train an ensemble ML classifier for identifying severe CAD, achieving a specificity of 92.7% and sensitivity of 84.4% during cross-validation. Integrating AI classifications into clinical practice bolstered CAD detection by 10%, enhancing inter-reader agreement, confidence, and sensitivity [40]. O’Driscoll et al. subsequently validated the model's high accuracy with an AUC of 0.93, while also delving into AI's potential for computing left ventricular ejection fraction (LVEF) and global longitudinal strain (GLS), thus refining stress echo interpretation [41]. The capability of the Treadmill Exercise Test (TET) to detect Obstructive Coronary Artery Disease (OCAD) is hindered by its limited sensitivity and specificity rates [42]. One primary limitation of TET is its unreasonably high rate of false positives [43]. A recent study introduced an ML system to enhance TET efficacy for CAD assessment. This study presented five models demonstrating diagnostic performance compared to conventional TET, focusing on ST-segment depression as a primary ECG finding. Ninety-three features were collected and narrowed down to 30 using feature selection methods. The most successful model, when compared to traditional TET, showed a 13% improvement in performance and a 20% increase in specificity with the addition of clinical features [44]. 

SPECT remains the primary method for assessing myocardial perfusion in CAD. The latest hardware features solid-state cadmium zinc telluride (CZT) detectors, which directly capture gamma rays from injected radiotracers. New collimator designs enhance photon sensitivity, while downsized scanners facilitate deployment. Optimized protocols and software emulate PET performance, offering reduced acquisition times, radiation exposure, improved resolution, and potential for quantification [45]. PET myocardial perfusion imaging has used a repurposed ResNet50 architecture, transfer learning, and data augmentation. This approach demonstrated how quantitative PET myocardial perfusion polar maps could predict MACEs at a two-year follow-up in a cohort of 1185 patients. Notably, the DL model, which excluded clinical or functional variables unlike other studies, exhibited superior discriminatory capacity, surpassing non-DL methods that integrated clinical variables, ventricular function, and absolute perfusion quantification (AUC = 0.90 vs. AUC = 0.85, p < 0.05) [46]. 

Future of AI in multimodal imaging

The advancement of AI methodologies for accurately predicting outcomes in cardiovascular disease [47], diagnosing CAD non-invasively [48], detecting malignant arrhythmias using wearable devices [49], and formulating a diagnosis, treatment strategies, and outcome prognostication for heart failure patients underscores AI's potential in shaping the future of cardiology [50]. The integration of AI advancements, the Internet of Things (IoT), and the promotion of precision medicine depicts a landscape where cardiology's trajectory heavily relies on these innovative digital technologies [50,51]. However, despite significant progress, ethical dilemmas surrounding the practical implementation of AI technologies in real-world medical settings still need to be addressed [48,52]. In conclusion, the intersection of cardiology and AI presents a realm brimming with possibilities for transforming patient care, ranging from improved diagnostics to personalized treatment approaches [53]. Nevertheless, addressing the ethical implications of AI integration in clinical practice is crucial as we embark on this transformative journey toward a digitally empowered future in cardiology [52,54]. 

Role for improvement

Despite significant advancements, several challenges must be addressed in integrating AI into multimodal cardiac imaging. Data standardization, algorithm validation, regulatory approval, and ethical considerations must be addressed to ensure AI technologies' safe and effective deployment in clinical practice. Collaborative efforts between clinicians, scientists, industry stakeholders, and regulatory bodies are essential to overcome challenges and unlock the full potential of AI in cardiac imaging. 

Limitations

The burgeoning role of AI in cardiac imaging heralds a new era of precision medicine, marked by enhanced diagnostic capabilities, personalized treatment strategies, and improved patient outcomes. As AI continues to evolve and integrate into clinical practice, its transformative impact on cardiac imaging is poised to redefine the landscape of cardiovascular healthcare. Models trained using data augmentation and transfer learning generalize well to different clinical centers, enhancing segmentation performance across diverse datasets. Explainable artificial intelligence (XAI) has emerged alongside AI advancements, focusing on tools and processes enabling humans to understand AI model workings and outcomes [55]. While classic ML models like linear regression and decision trees are more interpretable, deep learning models often outperform them, posing challenges in outcome interpretation and trust-building for clinical use [56]. Achieving a balance between model performance and interpretability is crucial for real-world deployment. XAI research addresses trustworthiness, transferability, fairness, and accessibility to ensure trustworthy AI systems [57]. Continued advancements in XAI will play a pivotal role in ensuring the adoption and effectiveness of AI technologies in healthcare. The possibilities seem endless with AI; however, efforts need to be targeted toward regulatory and quality control measures when dealing with primarily AI-generated data. For example, a checklist called the Proposed Requirements for Cardiovascular Imaging-Related Machine Learning Evaluation (PRIME) was introduced in 2020. This checklist covers seven key areas crucial for developing and reporting machine learning models, not just in cardiovascular imaging but potentially in other medical fields. It aims to ensure consistent and detailed reporting of ML studies while guarding against errors and biases arising from misunderstandings in this rapidly advancing field [58]. 

## Conclusions

In conclusion, despite emerging literature on ML-based AI in nuclear cardiac imaging suggesting that it could significantly improve cardiac risk prediction, it has certain limitations that warrant consideration. These include algorithm biases, data privacy concerns, interpretability issues, and the need for human oversight. Additionally, AI algorithms may only sometimes generalize well across diverse patient populations or clinical settings, necessitating ongoing validation and refinement. 

Integrating AI into clinical practice requires substantial changes, including heavy software integration, cultural shifts among healthcare professionals, and rigorous validation through prospective studies. ML models, which learn directly from data without predefined rules, present challenges such as opacity and potential biases, which could affect their reliability. Ensuring safe clinical use involves using representative data, maintaining strict quality control, and enhancing model interpretability. It is crucial to ensure that human oversight is maintained, adapting multimodal imaging to meet the specific needs of individual patients while considering their distinct biological, pathological, ethical, social, and personal traits.
